# Perceptions of Midwives on Pap Smear Tests during Pregnancy

**DOI:** 10.31557/APJCP.2020.21.10.3039

**Published:** 2020-10

**Authors:** Aderonke Elizabeth Abdul, Tshimangadzo Selina Mudau, Moleboge Antonia Chabedi

**Affiliations:** *Department of Health Care Sciences, Sefako Makgatho Health Sciences University, South Africa. *

**Keywords:** Pap smear, cancer screening, pregnancy, midwife, perceptions

## Abstract

**Purpose:**

The aim of the study was to explore and describe perceptions of midwives on Papanicolaou (Pap smear) test during pregnancy.

**Methods:**

The study used qualitative, exploratory type of design. A probability purposive sampling was used to sample 12 registered midwives based in gynaecological units in a public hospital in Tshwane District, South Africa. Open-ended questionnaires, field notes, and audio tape were used to collect data. Data analysis process involved grouping and categorization into themes and sub-themes.

**Results:**

This study showed that majority of midwives lacked scientific knowledge behind Pap smear test during pregnancy. Some of the participants could relate with the test and verbalized that there may be complications such as bleeding, which may lead to miscarriage. Conclusions: Findings showed that midwives were not performing Pap smear tests among pregnant women due lack of knowledge. This points out that Pap smear test is not prioritised as a secondary preventive tool at facility level. It is therefore recommended that refresher workshops be conducted at hospital level.

## Introduction

The burden of cervical cancer has been increasing despite several efforts to curb the disease. Globally cervical cancer incidence is at 569,000 causing more than 311,000 deaths (Saraiya, 2019). In 2018, cervical cancer was ranked as the fourth most common cause of cancer incidences and mortality in women worldwide (Arbyn, 2020). This type of cancer was the main cause of cancer-related deaths among women in eastern, middle, southern, and western Africa (Arbyn, 2020). Countries in the eastern, southern, and western Africa (namely, eSwatini, Malawi, Zambia, Zimbabwe, Tanzania, Burundi, Uganda, Lesotho, and South Africa) were found to have the highest cervical cancer incidences (ASIR >40 per 100,000) compared to western Asia, particularly Australia–New Zealand with ASIR <10 per 100,000. Accordingly, this provides the reason on the fact that nearly 84% of all cervical cancers and 88% of all deaths caused by cervical cancer occurred in lower-resource countries globally. In contrast, countries such as north America, northern and western Europe, Australia, New Zealand, northern Africa and western Asia have the lowest incidence rates. The low incidence among the northern Africa and western Asia countries could be attributed to societal practices around sexual activities leading to low prevalence of Human Papilloma Virus (HPV) (Arbyn, 2020). The incidence rate of sexually related infections (STI), such as Human Immuno-deficiency virus (HIV) is lower. On the other hand, countries such as north America, northern and western Europe, Australia and New Zealand provide extensive cytological screening programmes.

South Africa Human Papillomavirus (HPV) Information Centre (2019) reported an annual cervical cancer prevalence of approximately 12983 with 5595 deaths due to cervical cancer. In South Africa (SA) cancer of the cervix is the second most frequent among women (HPV information centre, 2019). A considerable number of women (20,2 millions) age 15 or more in RSA are at risk of cervical cancer (Bruni et al., 2018). Most recent data reports that approximately 3.2 % women aged 15-44 are harbouring HPV16/18 infection in a given time (HPV information centre, 2019). This could be the fact that the indicated age grouped could not benefit from the HPV vaccination programme was launched in 2014 in SA and early sexual engagement among most women (Botha and Dreyer, 2017). Most recently, the Gauteng Department of Health, the same Province where the study was conducted, launched new testing method of cervical smear-liquid base cytology (LBC) to reduce the incidence and prevalence rate through early detection and enrolment (Gauteng Department of Health, 2019). The report indicated that at least 52% of cervical screening rate 52% 2018/19. However, it is not stated if the increase is due to the new screening method or not.

Midwives have a horizontal approach to health care and public health; they are well-positioned to provide interventions at the individual and community level (Dahlen et al., 2019). This is to avoid missed opportunities among pregnant women (DoH, 2017). Pregnancy is a potential opportunity to counsel and educate pregnant women regarding the significance of Pap smear (Manikkam, 2016). Studies have confirmed that Pap smear test is safely done during pregnancy irrespective of gestational age (Botha and Dreyer, 2017; Heena et al, 2019; Manikkam, 2016). Progressively, several studies have confirmed positive intraepithelial lesions detected during pregnancy (Ncube et al., 2019, Manikkam, 2016). According to Rana et al. (2019) screening during pregnancy is very important, it focuses on detecting and preventing mother and child unfavourable occurrence and thus bring about early intervention. Remarkably, Priya and Shankar (2018) reported a statistical significance among on age at marriage (less than 20 years) and abnormal Pap smear results. The reported that nearly 86% of all squamous intraepithelial lesions (SIL) identified during pregnancy were classified as low-grade SIL. Therefore, secondary screening especially among women who had never been screen in five is vital to defeating cancer-related mortality especially among low-income groups (Saraiya, 2019).

## Materials and Methods

Based on the fact that no information was known on this topic in the study area, a qualitative, explorative design was used to explore and describe the understanding, information, views and of midwives (Denzin and Lincoln, 2019), working in the Gynaecology wards (Post-natal ward, Labour ward and Antenatal clinic) of a Public Hospital in Tshwane.

The study was conducted in the gynaecological section in a public hospital in Tshwane. This includes gynaecological and obstetrics wards with the Post-natal ward, Labour ward and Antenatal ward and clinic. Each of these wards had 10 registered midwives (n=40). The facility renders services for pregnant and non - pregnant women referred from other health facilities within the province. The population served is predominantly blacks from the middle to low-income class mostly from historically disadvantaged groups (University of Limpopo, 2016). A total of forty (40) participants were randomly selected for the study. A probability purposive sampling was done to access midwives allocated at the Gynaecology wards (Polit and Beck, 2017).

*Data collection*

The paper is part of the unpublished Master of Nursing (Abdul, 2020). University and Hospital ethical clearance was obtained before data collection at a public hospital in Tshwane. The period of data collection was five months whereby open-ended questionnaires, field notes, and audiotape were used to record responses from participants (Denzin and Lincoln, 2019; Polit and Beck, 2017). The rights of participants were explained and observed to ensure protection and privacy (Justus and Nangombe, 2016). An interview guide with central probing question was used to interview midwives for 30-45 minutes. Data saturation was reached at the 10th participants when no new information was obtained (Momberg et al., 2017). In the process, research trustworthiness was ensured through peer reviews where the data collection instrument was subjected to critique by two co-authors. Similarly, transcribed and coded data was critically reviewed by the two co-authors and the independent coder until consensus was reached. 

*Data analysis*

Data analysis was done following the eight Tesch’s open coding method of analysis (Creswell, 2017). Verbatim transcriptions of the voice recordings were done concurrently with data collection by the researcher. The audio recording for each interview was transcribed verbatim within 48 hours and analysed. The researcher read transcripts several times to be familiarized with the provided participants’ responses. This was followed by identification of themes. The researcher grouped common and related themes together as superordinate themes. Table 2 was drawn for quick judgement and decision of relating themes. The same process was followed by the independent coder and thus ensuring research results trustworthiness (Momberg et al., 2017). Comparing the researchers coding and that of independent coder lead to consensus.

## Results

The study explored perceptions of midwives regarding Pap smear test among pregnant women. An accessible population of 10 out of 40 midwives was interviewed. The interview guide included demographic profile (Table 1) of the participants and an opening question. Further probing questions were given to explore the perceptions further.

From the interviews three themes, seven categories and thirteen sub-categories emerged as indicated below (Table 2).

*Perceptions of Midwives on Pap smear test*

Most midwives responded positively regarding the Pap smear test on pregnant women. The majority (92%) of participants indicated that Pap smear test should be done during pregnancy especially those who have never done before. Eight percent showed negative attitude towards Pap smear tests during pregnancy. The negative attitudes were based fear of stimulating premature labour, miscarriages due to cervical irritation and infections. The results indicated participants’ lack of scientific knowledge regarding Pap smear test during pregnancy. One of the midwives said “*I think it should not be done on pregnant women because once they take the smear there is a possibility that the cervix will be stimulated and dilate leading to miscarriages*”. This kind of a response also showed that the midwives lack knowledge on the procedure of taking Pap smear. 

*Challenges experienced by midwives*

The challenges experienced by midwives on performing Pap smear on pregnant women were categorised as patient-centred, midwife-centred and health systems-centred (Table 2). The midwives reported that patient-centred challenges include; poor cooperation of pregnant mothers, and fear of complications from pregnant women. This was confirmed by statements such as; “*Most pregnant women are reluctant…Most women in general are reluctant to Pap smear …. they’ve got a perception that it’s painful and some people feel that it is degrading [sic] just to be checked down there……*” Another participant said the pregnant women refused Pap smear test because they believe it may cause abortion. 

Midwife-centred challenges on another hand, include participants indicating that they are afraid of the complications associated with Pap smear thus creating fear and anxiety towards the procedure. 

P3: “*I think we are scared to induce miscarriages because we think we might interfere with the cervical mucous then… cause preterm labours [sic]*”. 

The participants reported health systems challenges whereby there is a shortage of staff and material resources such as speculums, and litmus paper which end up hindering performance of Pap tests during pregnancy. 

P3“*………because of the workload … a lot of things that are done during ANC for pregnant women that are basic, plus … shortage of some equipment…*”.

Moreover, midwives showed limited knowledge of the exact period during which to perform Pap smear after delivery. It was raised that “*… I had that experience three days ago when you go to the clinic they do pap smear yes…. so I think that one is the best solution [sic]*”. This showed lack of knowledge on the recommended time to take Pap smear during post-natal period.

*Missed opportunity*

The majority of participants (85%) agreed that doing Pap smear test during pregnancy provides an opportunity to provide valuable service to those who may have missed such opportunity for various reasons. This was supported by some of the responses from the participants: 

P1“*Yes it can help, because you will find out that some women don’t attend clinic, the only time they attend clinic is when they are pregnant*”

 P2 “*…. you must use that opportunity to reduce that missed opportunities that we’re having…[sic]*”.

P4: “*Some people don’t attend clinic at all, so that might be only opportunity to have eh…to have a connection with the patient*’’

Midwives further recommended that providing in-service training and refresher courses for midwives could potentially remedy the situation. 

**Table 1:** Demographic Profile of Participants

Criterion	Characteristics	Frequency
Age	Below 40 year	9
	Above 40 year	1
Experience	Below 15 year	10
	Above 15 year	0
Qualification	Advance midwives	8
	Midwives	2
Race	Black	10
	White	0

**Table 2:** Themes, Categories and Sub-Categories

Themes	Categories	Sub-categories
Perceptions of midwives on Pap smear test on pregnant women	· Midwives factors- · Fear of complications	· Indifferent to Pap smear test during pregnancy (knowledge). · Fear of obstetric complications such as · Premature labor · Infection from premature rupture of membrane · Missed opportunity · Uncertainty of performing the procedural
	· Patient-related factors · Patient factors	· Attitude of pregnant women · Reluctance from pregnant woman · Pregnant women’s fear of complication Poor care-seeking behaviour
Challenges faced by Midwives in performing Pap smear test on pregnant women		
	· Midwife factors	· Lack of scientific knowledge · Shortage of staff · Work overload
	· Health systems shortcomings	· Lack of resources (human and material). · Work overload

## Discussion

Based on the demographic presentation, eight (8) out of ten (10) participants are advanced midwives, thus creating an expectation that adequate care is provided within the maternity section (Matlana and Lumadi, 2019). The findings however, indicated that, the midwives are not keen to perform Pap smear test during pregnancy due to lack of knowledge and negative attitudes among others. This corroborates with findings from Mudau and Human (2017) where experienced professional nurses could not use their specialised knowledge to provide care to children under five years of age in Makhado an area in the northern Province of Limpopo. The midwives’ non-compliance to policies regarding Pap smear testing defeats not only the country-specific objectives but the international policies as well. Missed opportunistic Pap Smear tests affects the attainment of the SDG goal 3 (good health and well-being) and 5 (gender equity) (United Nations, 2017). Moreover, based on the fact that HPV is the predominant cause of cancer of the cervix, Pap smear test during pregnancy can be opportune for early detection and treatment (Bruni et al., 2018; Julião, Savva-Bordalo and Lunet, 2017). It had been recommended in SA guideline (DoH, 2017) that health workers should use every opportunity to not only screen but further raise awareness for Pap Smear test in the lifetime of every woman. Given the prevalence of cancer of the cervix and the efforts made by the health authorities in RSA to capacitate midwives, it is expected of trained midwives to promotive and lead preventive measures to fight the scourge of cancer using opportunistic tests.

The results also revealed that midwives were aware of the fact that taking Pap smear test during pregnancy closes the gap of missed opportunities. These responses concur with the findings of Ncube et al., (2015) who reported that health workers reported various reasons for not taking Pap smear test among women in Jamaica. These findings were also reported in Ethiopia and the United States (Aweke et al., 2018) respectively. Even so, such missed opportunities are worrisome because cervical cancer is preventable disease if detected and treated early (Heena et al., 2019; Phaswana-Mafuya and Peltzer, 2017; Shashidhar and Jayasheelan, 2017). Moreover, such screening detects other sexually transmitted infections that can be harmful to the unborn baby. Additionally, the study setting is at a hospital that serves mainly black people from a lower and middle socio-economic status where access to better care may be limited (DoH2018/2019). This contradicts findings by Manikkam (2016) and Phaswana-Mafuya and Peltzer, 2017, who reported that women in the urban areas are well positioned to receive quality care than those in the rural areas. Generally, missed opportunities on cervical cancer screening can be compounded by the fact that SA does not have a national cancer screening programme which would speed up the process of achieving strategic goals of the Department while raising awareness among women (DoH, 2018; DoH 2019). 

Findings such as health professionals’ attitudes, patients’ inadequate knowledge, and lack of resources have been reported as contributions to the low-uptake of Pap smear test among women globally (Afzal et al., 2017; Godfrey et al., 2019; Manikkam 2016; Phaswana-Mafuya and Peltzer, 2017; DoH, 2017). When women are empowered with the knowledge they will probably take responsibility of their own health and seek for Pap smear test whenever it is due (DoH, 2017; Mingo et al., 2012). These findings concur with that of Heena et al., (2019) who found that health care workers at tertiary hospital in Saudi Arabia lacked knowledge on the importance of Pap smear screening which affected Pap smear uptake in the area. On the contrary, a study by Manikkam (2016) found that midwives and pregnant mothers were aware and receptive of Pap smear screening. Community education and awareness of the importance of early detections does not only curb the growth of cancer cells but also identifies sexual infections for early treatment (Ethirajan, Srinidhi and Jayashree, 2018) for screening, education and awareness that will stimulate care-seeking behaviour even after pregnancy.

Midwives mentioned both staff and material resources as challenges in providing Pap smear tests among pregnant women. The shortage of midwives is one of the challenges mentioned by participants in this study, this seems to have a major impact on the delivery of services and the unpleasant working conditions of midwives. The poor control of referral system is an element which causes extremely high workloads for midwives (Matlana and Lumadi, 2019). The lack of resources in this study correspond with findings by Julião et al., (2017) in Guinea-Bissau and Jamaica (Ncube et al., 2015). Sadly, it appears that shortage of human and material resources are becoming a norm in the African states. According to Moyimane, Matlala and Kekana (2019), medical equipment is essential for diagnosing, preventing and treating of patients, and thus, should be provided by health management to ensure quality patient care.

Some of the participants feared complications which was justified by Verma et al., (2017) and Gungorduk et al., (2016). Midwives’ indication of fear and uncertainty on the Pap smear test done during pregnancy in this study was anchored on the lack of knowledge and incompetency. Such an element of fear and misconception was also noted by Kumar et al., (2019). 

*Study Limitations*

The study excluded pregnant women and this had limitations in exploring care-seeking behaviour on Pap smear test among the women. Furthermore, statements from midwives that pregnant women are reluctant and refuse to perform Pap smear could have been validated. Document analysis to verify the number of women eligible for Pap smear test and identify missed opportunities. The results cannot be inclusive to all gynaecological and obstetric units in public hospitals in the Gauteng Province.
